# P-483. Late presentation of People Living With HIV in “Treat All” Era in Maputo City, Mozambique

**DOI:** 10.1093/ofid/ofae631.682

**Published:** 2025-01-29

**Authors:** Lucia Mabalane Chambal, Charlotta Nilsson, Elias Manjate, Corssino Tchavana, Vanda dos Muchangos, Orvalho Augusto, Esperanca Sevene Comiche

**Affiliations:** Eduardo Mondlane University, Maputo, Maputo, Mozambique; Karolinska Institutet, Stockholm, Stockholms Lan, Sweden; Eduardo Mondlane University, Maputo, Maputo, Mozambique; Manhiça Health Research Center (CISM), Maputo, Maputo, Mozambique; Eduardo Mondlane University, Maputo, Maputo, Mozambique; University of Washington, Seatle, Washington; Eduardo Mondlane University, Maputo, Maputo, Mozambique

## Abstract

**Background:**

Advanced HIV disease (AHD), defined as People Living with HIV with a CD4 cell count < 200 cells/mm^3^ or WHO clinical stage 3 or 4, increases the risk of severe opportunistic infections, hospitalizations, and death. Programmatic data from Mozambique showed a reduction in the prevalence of AHD at ART initiation from 73% to 37% during 2004-2014. Since 2016, Mozambique has adopted the “Treat all” strategy, and data on AHD at presentation for care is limited. We assess the prevalence of AHD in recently diagnosed HIV clients ART naïve.

**Figure:**
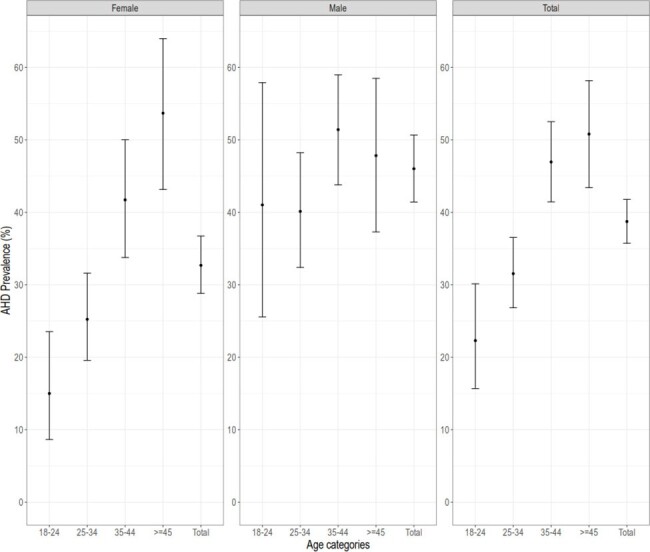

Relation between Age and Gender and AHD prevalece

**Methods:**

An analysis of baseline data of a prospective cohort study in newly HIV-diagnosed ART naïve at Mavalane Health Centre (MHC) located in a periurban area in Maputo City. April 2021-November 2022, all HIV-1 diagnosed ART naïve above 18 years of age were enrolled. A questionnaire was administered to assess risk factors for HIV infection, and CD4+ T cell counts and HIV viral load were determined.

**Results:**

A total of 1,025 PLHIV were enrolled, of whom 560 (55%) were females, median age of 35 (IQR: 30, 43) years, and mean BMI was 23.7 kg/m^2^ (SD: 4.48) with 8.1% under 18.5 kg/m^2^. The overall median CD4 + cell count was 252 (IQR: 140, 421) cells/mm^3^, with 46.0% (214/465, 95%CI: 41.4-50.7%) and 32.7% (183/560, 95%CI: 28.8-36.7%) of males and females, respectively, below 200 cells/mm^3^. There was an age-ascending trend (p-value: < 0.001) among females from 15.0% (95%CI: 8.6-23.5%) to 53.7% (95%CI: 43.2-64.0%). There was age dependency among males (p-value: 0.1887). The overall median log_10_ HIV-RNA was 5.12 (IQR: 4.67, 5.52), 4.41 (IQR: 3.63, 4.94) among AHD and non-AHD, respectively (p-value < 0.001).

**Conclusion:**

This study unveiled a high burden of AHD, showing that even in an era when countries are working on strategies for early HIV diagnosis and treatment to reduce the incidence of HIV and mortality associated with opportunistic infections, clients are still presenting in an advanced stage of the disease.

**Disclosures:**

**All Authors**: No reported disclosures

